# Retarding Progression of Myopia with Seasonal Modification of Topical Atropine

**Published:** 2010-04

**Authors:** Paul CS Lu, Jackie CL Chen

**Affiliations:** 1 Buddhist Tzu Chi General Hospital, Taipei, Taiwan; 2 Harvard Vision Center, Taipei, Taiwan; 3 Chang Gung Memorial Hospital, Chiayi, Taiwan

**Keywords:** Myopia, Atropine, Children, Taiwan

## Abstract

**Purpose:**

To investigate whether seasonal modification in the concentration of atropine drops is effective in retarding the progression of myopia.

**Methods:**

Two hundred and forty eyes of 120 healthy preschool- and school-age children in Chiayi region, Taiwan were recruited. The treatment group consisted of 126 eyes of 63 children who received atropine eye drops daily for one year and the control group included 114 eyes of 57 children who received nothing. The concentration of atropine eye drops was modified by seasonal variation as follows: 0.1% for summer, 0.25% for spring and fall, and 0.5% for winter. Refractive error, visual acuity, intraocular pressure (IOP), and axial length were evaluated before and after intervention.

**Results:**

Mean age was 9.1±2.8 years in the atropine group versus 9.3±2.8 years in controls (P=0.88). Mean spherical equivalent, refractive error and astigmatism were −1.90±1.66 diopters (D) and −0.50±0.59 D in the atropine group; corresponding values in the control group were −2.09±1.67 D (P=0.97) and −0.55±0.60 D (P=0.85), respectively. After one year, mean progression of myopia was 0.28±0.75 D in the atropine group vs 1.23±0.44 D in controls (P<0.001). Myopic progression was significantly correlated with an increase in axial length in both atropine (r=0.297, P=0.001) and control (r=0.348, P<0.001) groups. No correlation was observed between myopic progression and IOP in either study group.

**Conclusion:**

Modifying the concentration of atropine drops based on seasonal variation, seems to be effective and tolerable for retarding myopic progression in preschool- to school-age children.

## INTRODUCTION

Myopia is a common ocular disorder, and high or pathologic myopia (greater than 6 diopters) is associated with potentially blinding complications, such as macular degeneration, retinal detachment, glaucoma and cataracts.^1^–^4^ The prevalence of myopia in young adults in Taiwan, Hong Kong, and Singapore (60% to 80%) is higher than that in the United States and Europe (20% to 50%).^1^,^5^–^7^ Data released by the Taiwanese National Health Bureau in 2003 showed that the prevalence of myopia in preschool children aged 4–5 years, 5–6 years and 6–7 years was 11.36%, 15.18% and 18.84%, respectively.^8^ Corresponding figures based on data in 1991–1994 had been 4.79%, 5.68% and 7.34%, respectively.^8^ Therefore myopia appears to be an important and increasing national health issue in Taiwan.

The rate of myopia progression is highest in young children and the average age for stabilization of myopia is approximately 16 years.^1^ The earlier the onset of myopia, the more rapid its progression. Therefore, the value of any treatment capable of slowing down or arresting the progression of myopia cannot be overemphasized. The etiopathogenesis of myopia might include a genetic basis, excessive accommodation, prolonged near work, and proliferation of chondrocytes on the anterior margins of the sclera.^7^, ^9^–^12^

Among current therapeutic modalities used for treatment of myopia, atropine eye drops have been reported to retard myopia progression in three randomized clinical trials.^13^–^15^ Three different concentrations of atropine (0.1%, 0.25%, 0.5%) all significantly reduced myopia progression, with 0.5% atropine being the most effective.^15^ However, photophobia and poor compliance, especially with the highest concentration (0.5%), are drawbacks to such treatment.^16^ We, therefore, hypothesized that adjusting the concentration of atropine (i.e. 0.1%, 0.25%, and 0.5%), based on seasonal variation, sunlight intensity and myopia severity, may provide better physiological adaptation for preschool- and school-age children receiving myopia therapy particularly for subjects living close to the Tropic of Cancer such as the Chiayi region in Taiwan, or in areas of low latitude.

## METHODS

This study was a university-based, case-control study including 240 eyes of 120 healthy preschool and school-age children from October 2006 to December 2008 in Chiayi area, Taiwan. The target population included all pre-school and school aged students. Children with previous ocular trauma, traumatic cataracts, keratoconus, high myopia (−10 diopters [D] or higher), high hyperopia (>+3.0D), severe astigmatism (>−3.0D), ocular hypertension, or glaucoma were excluded. Subjects who received complete atropine treatment more than one year were included. The control subjects consisted of children who had received no medication for more than one year. The treatment group included 126 eyes of 63 children who received atropine eye drops, one drop in each eye for one year; 114 eyes of 57 children who received no treatment served as controls.

The concentration of atropine eye drops (0.1%, 0.25%, and 0.5%) was modified by seasonal variation, sunlight intensity and severity of myopia. In general, the 0.1% dilution was used in summer, the 0.25% dosage in spring and fall, and the 0.5% concentration in winter. For those who had myopia < −0.50D and were less than 7 years of age, 0.5% atropine was not applied. The frequency of instillation was reduced to twice weekly for very low myopes (−0.75 to 0 D). Anti-UV sunglasses were prescribed for subjects who had outdoor activities and progressive spectacles were given for children who had difficulty in the classroom.

Initial ocular examinations included uncorrected visual acuity (UCVA), measurement of refractive errors, best-spectacle corrected visual acuity (BSCVA), intraocular pressure (IOP) and axial length (AL) measurements, all of which were repeated during prescheduled visits every four to six weeks. Refraction was measured at baseline under cycloplegia (1% tropicamide) and refractions during the study or at final follow-up were obtained under the effect of atropine. We collected data at three-month intervals for statistical analysis.

Statistical analysis was performed using standard software (SPSS, version 11.01 for Windows, SPSS Corp., Chicago, USA). Student’s *t*-test and correlation coefficients were used for statistical analysis. Ninety-five percent confidence intervals were calculated to compare mean values. P<0.05 was considered as statistically significant.

## RESULTS

**Figure 1:**
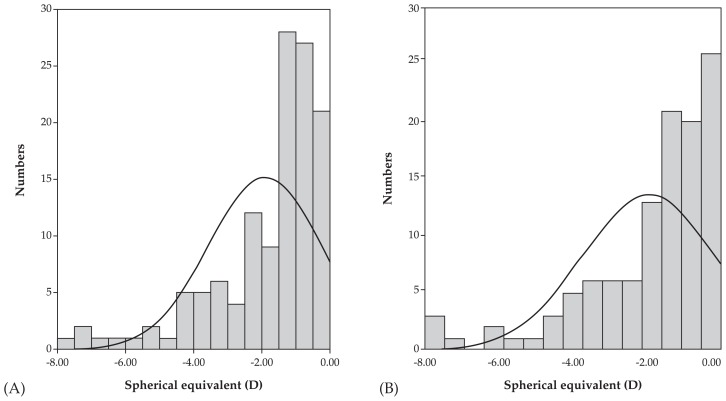
Distribution of myopia in the atropine (**A**, 126 eyes) and control (**B**, 114 eyes) groups at baseline.

**Figure 2:**
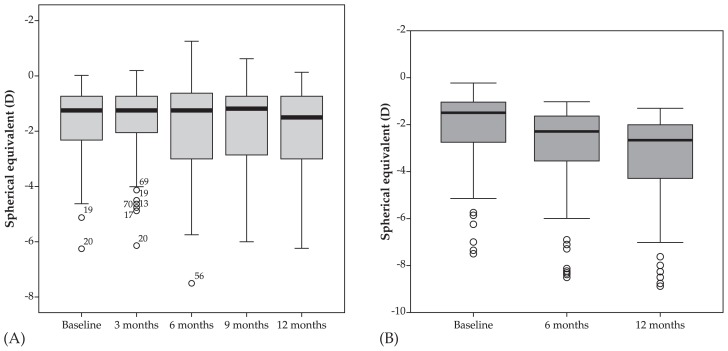
Changes in spherical equivalent refractive error in the atropine **(A)** and control **(B)** groups.

**Figure 3:**
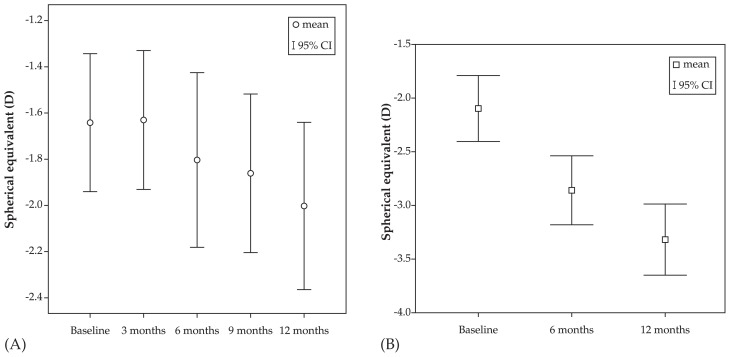
95% confidence intervals (CI) for spherical equivalent refractive error in the atropine **(A)** and control **(B)** groups.

Mean age was 9.1±2.8 (range, 4–16) years in the atropine group versus 9.3±2.8 (range, 4–16) years in the control group (P=0.88). At baseline, mean spherical equivalent (SE) refractive error was −1.90±1.66 (range 0 to −7.63) D and mean astigmatism was −0.50±0.59 (range, 0 to −2.50) D in the atropine group. Corresponding values in the control group were −2.09±1.67 (range, 0 to −7.50) D (P=0.969) and −0.55±0.60 (range 0 to −2.50) D (P=0.854), respectively (Figures 1A and 1B). After one year, mean myopia progression was 0.28±0.75 D versus 1.23±0.44 D in the treatment and control groups respectively (P < 0.001) (Table 1, Figures 2 and 3).

**Figure 4:**
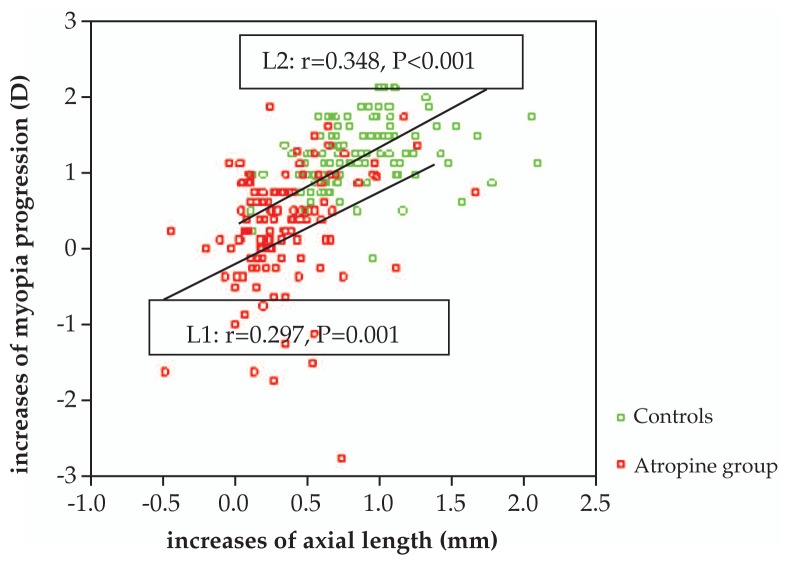
Correlation between progression of myopia and increase in axial length in the study groups.

Mean axial length at baseline was 23.78±0.94 mm and 23.92±0.83 mm in the atropine and control groups, respectively (P=0.129). Corresponding figures one year later were 24.12±0.99 mm and 24.78± 0.96 mm, respectively (P<0.001). Progression of myopia was significantly correlated with an increase in axial length in both the atropine (r=0.297, P=0.001) and control (r=0.348, P<0.001) groups as shown in Figure 4.

Baseline mean IOP was 17.39±3.97 and 17.89±3.48 mmHg (P=0.340) in the atropine and control groups, respectively which reached 18.06±3.37 and 18.14±3.29 mmHg (P=0.855) at final follow-up, respectively. The progression of myopia was not correlated with IOP increase in the atropine group (r=0.0023, P=0.907) or in the controls (r=0.001, P=0.907) as shown in Figure 5A. The progression of myopia was also not correlated with raw IOP values (r=−0.0015, P=0.923) as demonstrated in Figure 5B.

Adverse events such as papillae, follicles, subjective decrease in visual acuity, and difficulty in accommodation, were mild in severity. No serious adverse effects were reported.

## DISCUSSION

In an evidence-based review article, Saw et al^17^ reported no evidence supporting the effect of bifocal lenses, pressure-lowering eye drops, or soft contact lenses on retarding the progression of myopia. Only atropine eye drops were supported by levels B and I evidence (B, moderately important recommendation; I, strong evidence supporting recommendation) and were very cost-effective in this regard. Although treatment with 0.5% atropine was the most effective,^15^ children and parents usually refrained from such treatment owing to adverse effects, especially photophobia in strong sunlight. In our series, the mean annual progression of myopia (0.28±0.75D) with varying concentrations (0.1%, 0.25%, and 0.5%) of atropine based on seasonal variation and sunlight intensity, was greater than that of treatment with 0.5% atropine (0.04±0.63D), but less than that of 0.25% atropine (0.45±0.55D) or 0.1% atropine (0.47±0.91D) as reported by Shin and Chen.^15^ Therefore, adjusting the concentration of atropine is highly effective, especially in regions with full sunshine, such as areas close to the Tropic of Cancer or regions of low latitude.

Atropine, a muscarinic antagonist, acts by paralyzing accommodation and has a direct effect on scleral growth.^18^,^19^ Our results revealed that progression of myopia was significantly correlated with an increase in axial length. We thus might attribute the retardation of myopia to the slowed rate of scleral growth. Adverse effects of atropine include photophobia, subjectively decreased visual acuity, abnormalities of accommodation, macular degeneration, retinal toxicity, and cataract formation due to excessive ultraviolet light.^20^–^21^ We therefore prescribed anti-UV sunglasses and suggested wearing a sunscreen hat for patients with outdoor activities.

Siatkowski et al^22^ for the US Pirenzepine Study Group and Tan et al^23^ for the Asian Pirenzepine Study Group reported that 2% pirenzepine ophthalmic gel, a subtype selective M1 anti-muscarinic agent, used twice daily was associated with less increase in myopia, 0.26 D and 0.47 D respectively, over a 1-year treatment period as compared to their placebo-controlled groups who demonstrated mean increase in myopia of 0.53 D and 0.84 D, respectively. Chua et al^24^ in Singapore used 1% atropine to treat myopia and reported that myopia progression was only −0.28±0.92 D per year in the treated group as compared to −1.20±0.69 D per year in the placebo-controlled group. Fan et al^25^ in Hong Kong used topical 1% atropine eye ointment to treat myopia and reported that myopic progression was significantly less in the atropine group (+0.06±0.79 D) than in the control group (−1.19±2.48 D).

Most children or their parents suspend treatment in summer if high concentrations of atropine (0.5% or 1%) are prescribed. We probed other reasons patients gave up therapy which included irritation from eye drops, high expectations for myopia control, misunderstanding or incomplete education, and too much time spent for ocular examinations. By providing better physiologic adaptation, one may be able to improve such treatment. To improve compliance, we reduced the frequency of instillation to twice weekly for very low myopes (−0.75 to 0 D). Anti-UV sunglasses were prescribed for subjects who had outdoor activities and progressive spectacles (trifocal or bifocal) were given for those who had difficulty in the classroom or for reading. Trifocal lenses, not bifocals, had a better cosmetic appearance and was preferred by children.

It remains a myth that some subjects may achieve significant improvement or reduction in established myopia with atropine therapy. For example, in this study, myopia of −1.25 D regressed to emmetropia or −2.50 D improved to −1.0 D, after three to six months of atropine treatment and remained stabilized thereafter.

One previous study on intraocular pressure lowering eye drops showed no effect on retarding myopia progression.^26^ Our results also showed that progression of myopia was not correlated with IOP.

In our study axial length appeared to increase only minimally after continuous atropine therapy for more than one year. This effect may be attributed to increased scleral rigidity or slower scleral growth. We also have to consider the phenomenon of pseudomyopia that is the power difference before and after cycloplegia in the clinic, because the mydriatic agent might not completely paralyze accommodation. More cases are needed to elucidate this issue.

We hypothesize the “trigger theory” such that children born by high myopic parents are like a pistol loaded with bullets (the genome) that might not do any harm if not triggered (by risk factors); but a lot of damage (complications) might follow if the triggers are pulled again and again. Atropine acts as a viscous medium to dampen or slow down such bullets thus reducing the damage.

In Singapore, Tong et al^27^ reported that after stopping treatment, eyes treated with atropine demonstrated higher rates of myopia progression as compared to eyes treated with placebo. However, absolute myopia progression after 3 years was significantly lower in the atropine group as compared to controls. We thus strongly suggest that myopia should be treated intensively and as early as possible in order to avoid late complications caused by high myopia. The current study demonstrated that by improving compliance to the medication and reducing complaints of photophobia, the modified use of atropine concentrations of 0.1%, 0.25%, and 0.5%, based on seasonal variation and severity of myopia, effectively delayed myopia progression in preschool and school-age children. Therefore in subjects with low to moderate myopia, atropine appears to significantly retard the progression of myopia. Whether pathologic or extreme myopia could be prevented by the use of atropine requires a large scale longitudinal study.

## Figures and Tables

**Figure 5A:**
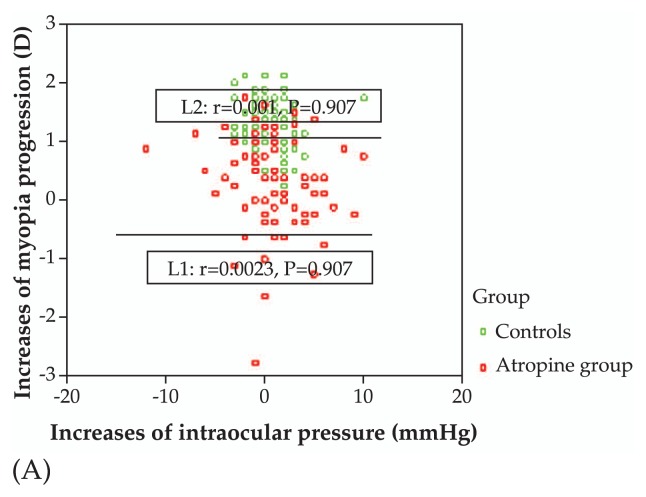
Correlation between progression of myopia and increase in intraocular pressure in the study groups.

**Figure 5B:**
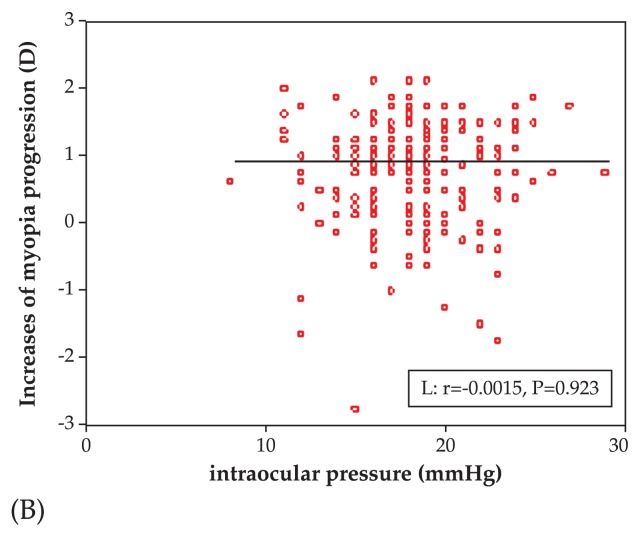
Correlation between progression of myopia and intraocular pressure in all subjects.

**Table 1:** Spherical equivalent refractive error (diopters) in the study groups

	Mean	SD	Min	Max
Treatment group				
Baseline	−1.90	1.66	−7.63	0

3 months	−1.92	1.68	−7.63	0.19

6 months	−1.98	1.84	−8.38	1.25

9 months	−2.04	1.80	−8.38	0.63

12 months	−2.17	1.88	−8.25	0.75

Controls				
Baseline	−2.09	1.67	−7.50	−0.250

6 months	−2.86	1.76	−8.50	−1.00

12 months	−3.32	1.79	−8.88	−1.25

SD, standard deviation; Min, minimum; Max, maximum.

## References

[1] Tan NW, Saw SM, Lam DS, Cheng HM, Rajan U, Chew SJ (2000). Temporal variations in myopia progression in Singaporean children within an academic year. Optom Vis Sci.

[2] Mitchell P, Hourihan F, Sandbach J, Wang JJ (1999). The relationship between glaucoma and myopia: the Blue Mountains Eye Study. Ophthalmology.

[3] Pierro L, Camesasca FI, Mischi M, Brancato R (1992). Peripheral retinal changes and axial myopia. Retina.

[4] Fan DS, Lam DS, Lam RF, Lau JT, Chong KS, Cheung EY (2004). Prevalence, incidence, and progression of myopia of school children in Hong Kong. Invest Ophthalmol Vis Sci.

[5] Lin LL, Shih YF, Tsai CB, Chen CJ, Lee LA, Hung PT (1999). Epidemiologic study of ocular refraction among schoolchildren in Taiwan in 1995. Optom Vis Sci.

[6] Katz J, Tielsch JM, Sommer A (1997). Prevalence and risk factors for refractive errors in an adult inner city population. Invest Ophthalmol Vis Sci.

[7] Saw SM, Katz J, Schein OD, Chew SJ, Chan TK (1996). Epidemiology of myopia. Epidemiol Rev.

[8] Bureau of Health Promotion, Department of Health, R.O.C., (Taiwan) www.bhp.doh.gov.tw.

[9] Chen JC, Schmid KL, Brown B (2003). The autonomic control of accommodation and implications for human myopia development: a review. Ophthalmic Physiol Opt.

[10] Yap M, Wu M, Liu ZM, Lee FL, Wang SH (1993). Role of heredity in the genesis of myopia. Ophthalmic Physiol Opt.

[11] Farbrother JE, Kirov G, Owen MJ, Pong-Wong R, Haley CS, Guggenheim JA (2004). Linkage analysis of the genetic loci for high myopia on 18p, 12q, and 17q in 51 U.K. Families. Invest Ophthalmol Vis Sci.

[12] Wang IJ, Shih YF, Tseng HS, Huang SH, Lin LL, Hung PT (1998). The effect of intravitreal injection of atropine on the proliferation of scleral chondrocyte in vivo. J Ocul Pharmacol Ther.

[13] Bedrossian RH (1979). The effect of atropine on myopia. Ophthalmology.

[14] Chou AC, Shih YF, Ho TC, Lin LL (1997). The effectiveness of 0.5% atropine in controlling high myopia in children. J Ocul Pharmacol Ther.

[15] Shih YF, Chen CH, Chou AC, Ho TC, Lin LL, Hung PT (1999). Effects of different concentrations of atropine on controlling myopia in myopic children. J Ocul Pharmacol Ther.

[16] Chiang MF, Kouzis A, Pointer RW, Repka MX (2001). Treatment of childhood myopia with atropine eyedrops and bifocal spectacles. Binocul Vis Strabismus Q.

[17] Saw SM, Shih-Yen EC, Koh A, Tan D (2002). Interventions to retard myopia progression in children. Ophthalmology.

[18] Tigges M, Iuvone PM, Fernandes A, Sugrue MF, Mallorga PJ, Laties AM (1999). Effects of muscarinic cholinergic receptor antagonists on postnatal eye growth of rhesus monkeys. Optom Vis Sci.

[19] Lind GJ, Chew SJ, Marzani D, Wallman J (1998). Muscarinic acetylcholine receptor antagonists inhibit chick scleral chondrocytes. Invest Ophthalmol Vis Sci.

[20] Goss DA (1982). Attempts to reduce the rate of increase of myopia in young people - a critical literature review. Am J Optom Physiol Opt.

[21] Luu CD, Lau AM, Koh AH, Tan D (2005). Multifocal electroretinogram in children on atropine treatment for myopia. Br J Ophthalmol.

[22] Siatkowski RM, Cotter S, Miller JM, Scher CA, Crockett RS, Novack GD, US Pirenzepine Study Group (2004). Safety and efficacy of 2% pirenzepine ophthalmic gel in children with myopia: a 1-year, multicenter, double-masked, placebo-controlled parallel study. Arch Ophthalmol.

[23] Tan DT, Lam DS, Chua WH, Shu-Ping DF, Crockett RS, Asian Pirenzepine Study Group (2005). One-year multicenter, double-masked, placebo-controlled, parallel safety and efficacy study of 2% pirenzepine ophthalmic gel in children with myopia. Ophthalmology.

[24] Chua WH, Balakrishnan V, Chan YH, Tong L, Ling Y, Quah BL (2006). Atropine for the treatment of childhood myopia. Ophthalmology.

[25] Fan DS, Lam DS, Chan CK, Fan AH, Cheung EY, Rao SK (2007). Topical atropine in retarding myopic progression and axial length growth in children with moderate to severe myopia: a pilot study. Jpn J Ophthalmol.

[26] Jensen H (1988). Timolol maleate in the control of myopia. A preliminary report. Acta Ophthalmol Suppl.

[27] Tong L, Huang XL, Koh AL, Zhang X, Tan DT, Chua WH (2009). Atropine for the treatment of childhood myopia: effect on myopia progression after cessation of atropine. Ophthalmology.

